# A case of peristomal pyoderma gangrenosum with histological features of pseudoxanthoma elasticum

**DOI:** 10.1016/j.jdcr.2024.11.032

**Published:** 2024-12-09

**Authors:** Kelly M. Kimball, Matthew J. Franklin

**Affiliations:** Department of Dermatology, OhioHealth Riverside Methodist Hospital, Columbus, Ohio

**Keywords:** dermatopathology, neutrophilic dermatosis, pseudoxanthoma elasticum, pyoderma gangrenosum

## Introduction

Pseudoxanthoma elasticum (PXE) is an inherited genodermatosis that causes extensive degeneration of elastic tissue.^1^ The pathogenesis of PXE is the consequence of a mutation in the *ABCC6* gene resulting in the abnormal calcification of connective tissue.^2^^,^^3^ There are several published cases that describe PXE-like histological changes in a number of inflammatory conditions without overt clinical manifestation of PXE. Such reported cases include lipodermatosclerosis, granuloma annulare, lichen sclerosus, morphea profunda, erythema nodosum, septal panniculitis, basal cell carcinoma, and fibrosing dermatitis.^4^ Herein, we present a case of peristomal pyoderma gangrenosum (PG) with histological features reminiscent of PXE, which, to our knowledge, has not been previously reported.

## Case report

**Fig 1:**
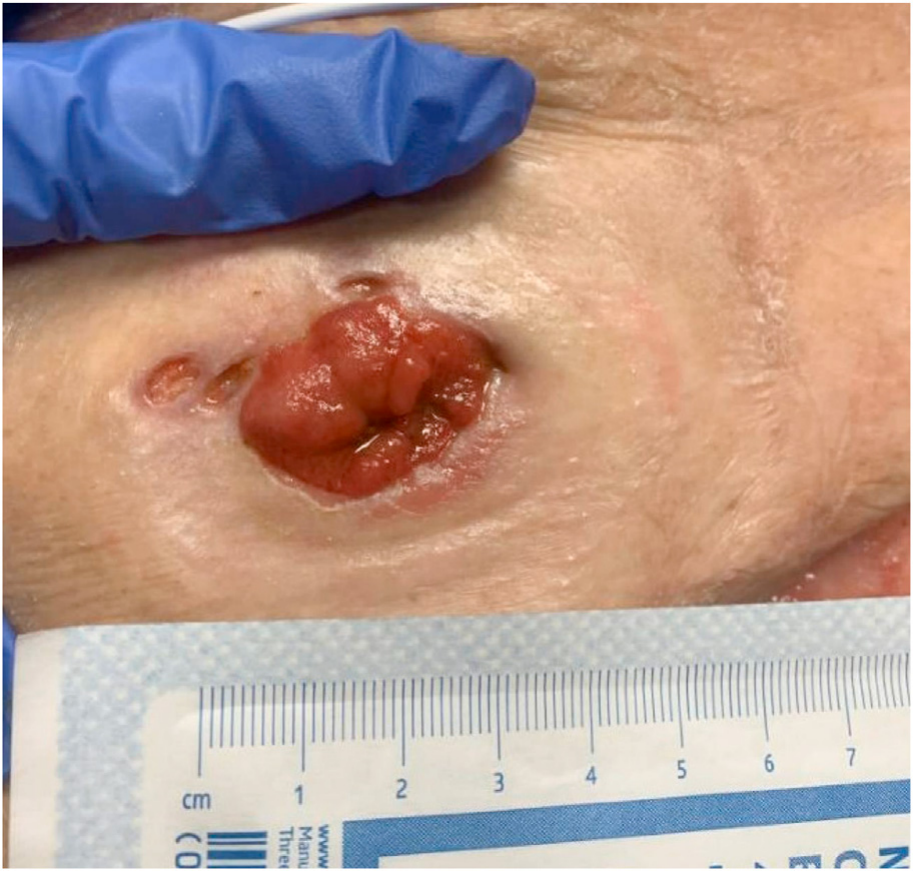
Physical examination revealed multiple peristomal ulcers with erythematous, undermined borders.

**Fig 2:**
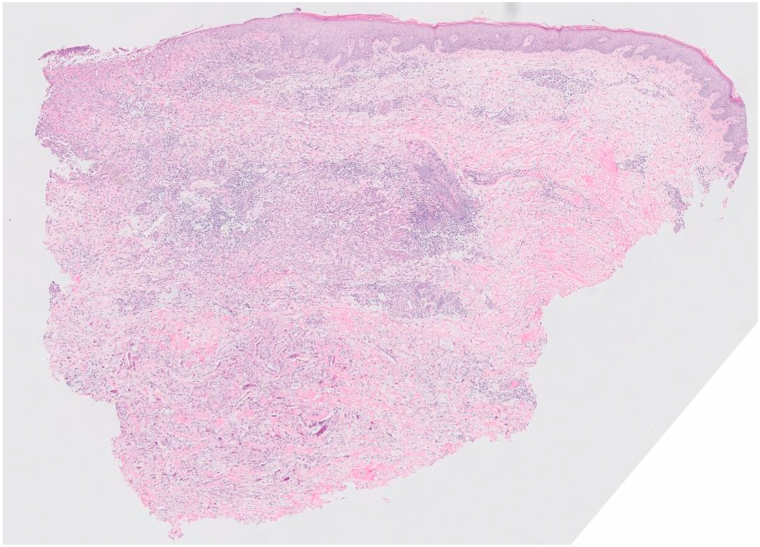
Low power (hematoxylin and eosin 20×) examination of sections from an ulcer’s edge demonstrates a diffuse dermal infiltrate with interstitial edema and basophilic bodies within the deep reticular dermis.

**Fig 3:**
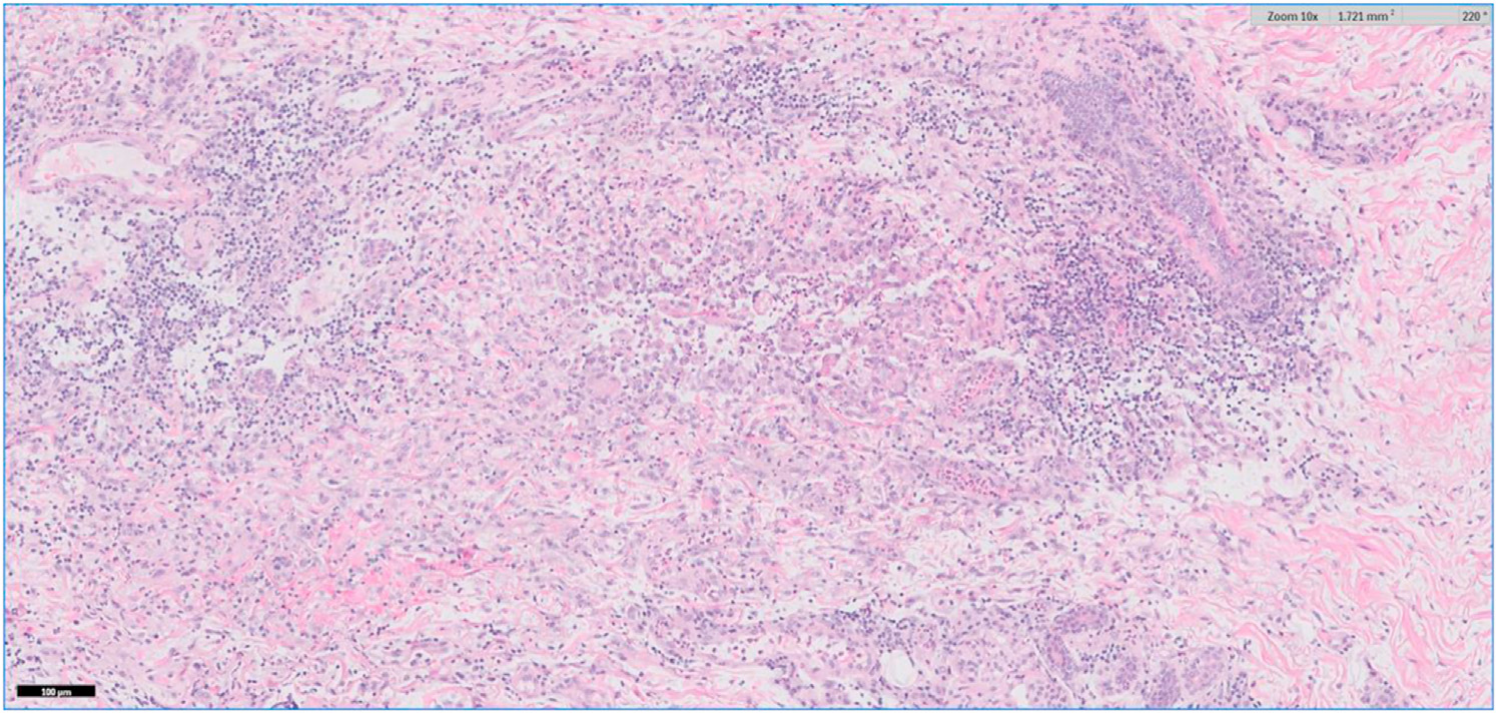
Higher power magnification (hematoxylin and eosin 100×) demonstrates interstitial edema and a mixed inflammatory infiltrate consisting of lymphocytes, histiocytes, and neutrophils.

**Fig 4:**
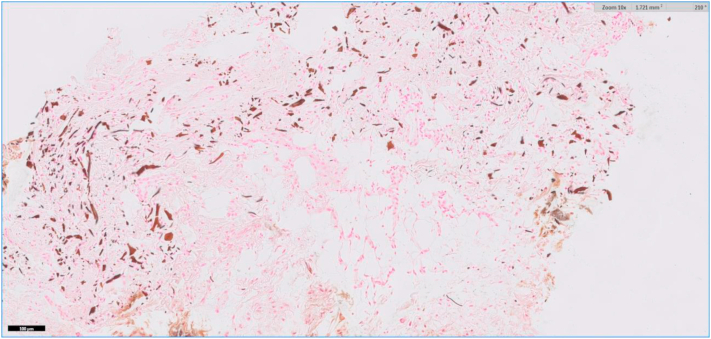
A von Kossa stain highlights calcium salts bound to dermal elastic fibers (100×).

A 64-year-old female with a past medical history significant for ulcerative pancolitis treated with infliximab and ostomy, atrial fibrillation, and stage III chronic kidney disease presented to the emergency department with a chief complaint of dizziness. She was subsequently admitted for hypovolemia from high ostomy output. Her hospital course was complicated by sepsis and acute respiratory failure requiring intubation and admission to the medical intensive care unit. On the third day of admission, dermatology was consulted due to concern for a desquamating rash. At that time, her serum calcium was decreased at 8.2 mg/dL and her serum creatinine was 1.64 mg/dL. She did not demonstrate any other signs of systemic infection. Her most recent lipid panel showed no abnormalities. Physical examination revealed multiple peristomal ulcers, as seen in Fig 1. The clinical differential diagnosis included peristomal PG and calciphylaxis. Biopsy revealed focal ulceration of the epidermis with a fibroinflammatory crust (Fig 2). In the dermis, a diffuse mixed inflammatory infiltrate composed of lymphocytes, histiocytes, and neutrophils with karyorrhectic debris was observed (Fig 3). Also identified in the dermis were basophilic and fragmented elastic fibers, reminiscent of dystrophic elastic fibers as seen in PXE. Gomori methenamine silver and acid-fast bacilli stains were negative for fungal organisms and acid-fast bacilli, respectively. A von Kossa stain demonstrated calcified elastic fibers in the dermis (Fig 4). The patient responded promptly to systemic corticosteroids. As a result, tissue cultures were not obtained. Utilizing Delphi criteria published in 2018 (ie, neutrophilic infiltrate, exclusion of infection, history of inflammatory bowel disease, clinical examination findings, and response to treatment with corticosteroid), a diagnosis of peristomal PG with PXE-like histologic features was rendered. The patient was not on penicillamine therapy, did not have a family history of PXE, and did not have any physical exam stigmata to suggest PXE. Because of this and due to the lack of availability at our community hospital, genetic studies were not performed.

## Discussion

Peristomal PG is a subtype of PG and is thought to represent a pathergic response to trauma secondary to mechanical irritation to the skin and/or fecal contamination, both of which are frequently seen in the context of stomas in patients with inflammatory bowel disease such as Crohn disease and ulcerative colitis.^5^ Histologically, PG demonstrates a mixed dermal infiltrate with neutrophil predominance, edema, and on occasion, leukocytoclastic vasculitis.^5^ The biopsy of our patient’s peristomal ulcer showed a mixed dermal inflammatory infiltrate consisting of neutrophils, lymphocytes, and histiocytes as well as calcified elastic fibers in the dermis that resembled those seen in PXE. This is in contrast to PXE-like inflammatory changes feature degenerative elastic fibers found deeper in the dermis or even subcutis, the latter being unique to PXE-like changes occurring in inflammatory states such as calciphylaxis.^6^ Given the patient’s chronic kidney disease and new-onset ulcerations, calciphylaxis was also included in the initial differential diagnosis; however, given the ulcers’ prompt improvement with corticosteroids, this diagnosis was excluded and a diagnosis of PG was further supported.^5^ Notably, this patient's ulcerative pancolitis was being treated with infliximab, which has been demonstrated to be an effective treatment for PG.^7^

To our knowledge, this report is the first to describe a case of PG in association with PXE-like histologic features.

## Conflicts of interest

None disclosed.
